# Prevalence and correlates of low-grade systemic inflammation in adult psychiatric inpatients: An electronic health record-based study

**DOI:** 10.1016/j.psyneuen.2018.02.031

**Published:** 2018-05

**Authors:** Emanuele F. Osimo, Rudolf N. Cardinal, Peter B. Jones, Golam M. Khandaker

**Affiliations:** a Department of Psychiatry, University of Cambridge, Cambridge, England, UK; b Cambridgeshire and Peterborough NHS Foundation Trust, Cambridge, England, UK

**Keywords:** Psychosis, Depression, CRP, Inflammation, White cell count, Immunopsychiatry

## Abstract

Low-grade inflammation is a risk factor for depression, psychosis and other major psychiatric disorders. It is associated with poor response to antidepressant and antipsychotics, and could potentially be a treatment target. However, there is limited data on the prevalence of low-grade inflammation in major psychiatric disorders, and on the characteristics of patients who show evidence of inflammation. We examined the prevalence of low-grade inflammation and associated socio-demographic and clinical factors in acute psychiatric inpatients.

An anonymised search of the electronic patient records of Cambridgeshire and Peterborough NHS Foundation Trust was used to identify patients aged 18–65 years who were hospitalised between 2013 and 2016 (inclusive). We excluded patients on antibiotics or oral steroids, or with missing data. Inflammation was defined using serum C-reactive protein (>3 mg/L) or total white cell count (>9.4 × 10^9^/L) as measured within 14 days of admission.

Out of all 599 admissions, the prevalence of inflammation (serum CRP >3 mg/L) in the ICD-10 diagnostic groups of psychotic disorders (F20–29), mood disorders (F30–39), neurotic disorders (F40–48) and personality disorders (F60–69) was 32%, 21%, 22% and 42%, respectively. In multivariable analyses, low-grade inflammation was associated with older age, black ethnicity, being single, self-harm, diagnoses of schizophrenia, bipolar disorder, current treatments with antidepressants, benzodiazepines, and with current treatment for medical comorbidities.

A notable proportion of acutely unwell psychiatric patients from all ICD-10 major diagnostic groups show evidence of low-grade inflammation, suggesting inflammation may be relevant for all psychiatric disorders.

## Highlights

• Up-to 40% acute psychiatric inpatients show evidence of low-grade inflammation.
• Low-grade inflammation is present in all ICD-10 major psychiatric diagnostic groups.
• Older age increases inflammation, while being married appears to be protective.
• Inflammation is associated with diagnosis of schizophrenia and bipolar disorder.
• Inflammation is associated with prescribed antidepressants and medical comorbidity.

## 1 Introduction

Major psychiatric disorders such as depression, psychosis and anxiety are associated with low-grade systemic inflammation, as reflected by elevated concentrations of pro-inflammatory cytokines, e.g. interleukin-6, and acute phase proteins, e.g. C-reactive protein (CRP), in peripheral blood during acute illness (Dickerson et al., 2016; Goldsmith et al., 2016; Howren et al., 2009; Maes, 1999; Upthegrove et al., 2014; Vogelzangs et al., 2013). Population-based longitudinal studies have shown that elevated concentrations of inflammatory markers during pregnancy or in childhood are associated with higher risk of developing symptoms of depression, psychosis and mania subsequently in adulthood (Canetta et al., 2014; Dalman et al., 2008; Hayes et al., 2017; Khandaker et al., 2014; Khandaker et al., 2012; Khandaker et al., 2013; Liang and Chikritzhs, 2012; Metcalf et al., 2017), suggesting that low-grade systemic inflammation may contribute to the development of major psychiatric disorders. Further evidence for a role of inflammation in psychiatric disorders comes from treatment studies. Meta-analyses of clinical trials indicate that anti-inflammatory drugs may have antidepressant effects. Non-Steroidal Anti-inflammatory Drugs (NSAIDs) and cytokine inhibitors given as adjuncts to antidepressants improve depressive symptoms in patients with depression (Köhler et al., 2014). Anti-cytokine drugs, which target inflammation more specifically, reduce the severity of depressive symptoms in patients with chronic inflammatory illness independently of improvements in physical illness (Kappelmann et al., 2016). However, evidence for the efficacy of anti-inflammatory treatment for psychotic disorders is less clear-cut. While some studies did not find an effect (Girgis et al., 2017; Miller et al., 2016; Nitta et al., 2013), adjunctive treatment with aspirin may be beneficial for psychosis (Sommer et al., 2013). Minocycline, a tetracyclic antibiotic, may improve negative symptoms and cognition in the early stages of schizophrenia (Chaudhry et al., 2012; Solmi et al., 2017).

It is likely that low-grade inflammation or anti-inflammatory drugs will be relevant for a subset of patients, because not all individuals with a major psychiatric disorder show evidence of inflammation. However, the prevalence of low-grade inflammation in patients with psychiatric disorders and clinical characteristics of patients who show evidence of inflammation is poorly understood. Previous studies have often compared concentrations of inflammatory markers between cases and non-cases, but there is limited data from clinical samples as to what proportion of patients with different psychiatric disorders show evidence of low-grade systemic inflammation. The proportion of depressed patients with elevated CRP ranges from 19 to 47% according to previous studies, but studies based on acutely unwell inpatients are scarce (Cizza et al., 2009; Raison et al., 2013; Rethorst et al., 2014; Uher et al., 2014; Wysokiński et al., 2015). A large, population-based study from Denmark reported that about one third of patients with a history of hospitalisation for depression show evidence of low-grade systemic inflammation, defined as a serum CRP level >3 mg/L (Wium-Andersen et al., 2013). However, data on the prevalence of inflammation in other patient groups is limited.

A better understanding of psychiatric patients who present with evidence of inflammation is necessary because inflammation is thought to contribute to treatment resistance. Higher pre-treatment levels of IL-6 predict a poorer response to antidepressants (Lanquillon et al., 2000; Maes et al., 1997) and antipsychotics (Lin et al., 1998; Mondelli et al., 2015) in patients with depression and schizophrenia, respectively. Higher baseline CRP levels are associated with improvements in depressive symptoms in treatment resistant depression treated with infliximab, an anti-TNF-α monoclonal antibody (Raison et al., 2013).

The aims of this study were to examine the prevalence of low-grade systemic inflammation in acutely unwell psychiatric inpatients from all major ICD-10 diagnostic groups, and to elucidate the demographic and clinical factors associated with inflammation in this population. We compared psychiatric patients with and without low-grade systemic inflammation on a number of characteristics, including socio-demographic factors, primary diagnosis, prescribed medication, medical comorbidity, self-harm, alcohol misuse and length of admission. We repeated the analyses by defining inflammation using total white cell count (WBC) to check the robustness of associations observed using CRP.

## 2 Material and methods

### 2.1 Setting

We carried out an anonymised search of the electronic patient records of the UK National Health Service (NHS) Cambridgeshire and Peterborough NHS Foundation Trust (CPFT) to identify patients hospitalised between 2013 and 2016 (inclusive). All patients had been hospitalised to this mental health hospital for the treatment of a psychiatric disorder. Patient records were de-identified using the Clinical Record Interactive Search (CRIS) for secondary research (Fernandes et al., 2013), and transferred into a research database with NHS and institutional approvals (UK NHS National Research Ethics Service reference 12/EE/0407). All patients who are admitted to CPFT acute hospitals are offered blood testing for CRP and WBC as per hospital protocol.

### 2.2 Sample selection and electronic search procedure

We searched the CRIS database for records meeting the following inclusion criteria: a) patients admitted to inpatient beds between 2013 and 2016, inclusive; b) aged 18–65; c) had a recorded ICD-10 psychiatric diagnosis (F01–F99); d) a blood test result for CRP or for WBC had been recorded on the electronic medical notes system within 14 days of admission. Exclusion criteria were: a) patients taking antibiotics (proxy for a current acute infection), b) patients on oral steroids. Patients on NSAIDs were not excluded as the presence of a chronic inflammatory condition is a key confounder and we wanted to be able to correct for it.

### 2.3 Extraction and coding of categorical information

For each patient, we extracted the following categorical information directly from the database: date of birth/age, sex, ethnicity, marital status, admission date, current ICD-10 diagnoses, and length of index admission. When a patient was admitted more than once in a six-month period, the first admission was used to avoid over-representation of patients with recurrent admissions (see also Supplementary Figs. 1 and 2). Data extracted from CRIS included diagnostic codes which could have been assigned to that patient at any time in the past according to the 10th Revision of the World Health Organization International Statistical Classification of Diseases and Related Health Problems (ICD-10). Treating clinicians assigned the diagnoses, which were recorded by clinicians or administrative staff.

### 2.4 Extraction and coding of CRP and WBC data, and definition of low-grade inflammation

A custom-built natural language processing software was used to extract numerical data relating to blood inflammatory markers. We extracted all available data relating to CRP and WBC. Only entries where CRP or WBC were recorded were kept for further analysis. The method for data extraction was accurate and reliable as measured by *recall* (probability of retrieving a record given it was relevant) and *precision* (probability of a record being relevant, given it was retrieved) statistics (see Supplementary Methods for procedure of calculating these statistics). Blood samples from patients admitted in Cambridge or Peterborough were tested in different labs, using assays with different sensitivity. According to the US Centers for Disease Control and Prevention and American Heart Association guidelines CRP levels over 3 mg/L is considered to be high (Pearson et al., 2003; Ridker, 2003); such levels are associated with increased risks of cardiovascular disease (Koenig et al., 1999) and psychiatric illnesses such as schizophrenia (Metcalf et al., 2017) in population-based studies. For the purpose of this study, we have defined low-grade inflammation as a serum CRP level >3 mg/L. This is because the hospital laboratory only reported an exact value for CRP if it was equal or over 4 mg/L; levels below this threshold were reported as ≤3 mg/L (see Supplementary Methods for further details). Inflammation was coded as a binary variable: not inflamed (CRP ≤3 mg/L) or inflamed ( 3 mg/L). For analyses using total WBC, we selected a cut-off value of 9.4 × 10^9^/L to define inflammation. This cut-off represents the third quartile of the distribution of WBC in our sample. This threshold is lower than the most common UK upper reference value for total WBC (11 × 10^9^/L). Therefore, our approach captured subjects with low-grade inflammation rather than those with very high inflammation.

### 2.5 Data on prescribed medications including medical comorbidity

A list of medications prescribed within +/−3 months of current admission was extracted using the General Architecture for Text Engineering (GATE) software (Cunningham et al., 2013). Medications were manually classified in drug classes (antipsychotics, antidepressants, benzodiazepines and sleep inducers, mood stabilisers, medication for medical comorbidities, NSAIDs and pain control medication, antibiotics – exclusion criterium, oral steroids – exclusion criterium). Antipsychotic medications were further divided into sub-classes (typical and atypical). Current prescriptions for an anti-hypertensive, diuretic, anti-diabetic, statin, anti-aggregant, anti-coagulant or medication for the management of dyslipidaemias were used as proxy for the presence of common, chronic medical illness. See the Supplementary Methods for further details about medication data coding.

### 2.6 Main psychiatric diagnosis

Many patients had more than one recorded diagnosis. We used a hierarchical method to assign one “main diagnosis” per patient as follows: organic mental disorder > psychotic disorder > mood disorder > anxiety disorder > personality disorder > other psychiatric diagnosis. Presence of a diagnosis in an earlier category trumped diagnosis in subsequent categories, i.e., if a patient had recorded diagnoses of both a psychotic disorder and an anxiety disorder, psychotic disorder was chosen as main diagnosis.

### 2.7 Statistical analysis

We calculated the prevalence of inflammation, defined as CRP >3 mg/L, for each major ICD-10 diagnostic group. We used logistic regression to calculate the odds ratios (ORs) and 95% confidence intervals (CIs) for inflammation (CRP >3 mg/L) for the following factors: age, sex, ethnicity, marital status, main diagnosis, self-harm, alcohol abuse, medical comorbidities, current medications, length of admission. All predictors were coded as categorical variables. Age was converted to a categorical variable using the 25th, 50th and 75th centiles as cut-offs, which correspond to age 28, 39 and 49 years respectively. Length of admission was converted to binary as above or below median (13 days). The ORs were calculated using the following groups as reference: female sex, white British ethnicity, single status, age <28, “other” diagnosis, “short” admission (<13 days). The same procedure was followed for analyses where inflammation was defined as total WBC >9.4 × 10^9^/L.

In addition, an independent sample *t* test was used to compare mean values for continuous variables between groups with and without inflammation (e.g. age, length of admission); a Chi-squared test was used for categorical variables (e.g. sex, marital status and ethnicity). We tested the association between CRP (binary variable) and total WBC (continuous variable) using logistic regression; high CRP (>3 mg/L) was the dependent variable, and WBC was the independent variable. All statistical analyses were performed in R (R Core Team, 2017). Plots were made using ggplot2 (Wickham, 2009), using the Cairo R graphics device (Urbanek and Horner, 2005).

## 3 Results

### 3.1 Samples

The electronic search yielded data on 6731 admissions for patients of any age to CPFT inpatient facilities between 2013 and 2016 (inclusive). After applying inclusion and exclusion criteria, our analytic sample comprised 599 admissions with data on CRP (546 unique patients). Admissions with data on WBC were 1072 (978 unique patients). There were no differences in sex, ethnicity, or age distribution between patients who had available blood results (analytic sample), and those who didn’t, however the analytic sample was relatively impoverished in married patients (see Supplementary Table 1). For CRP data, recall was 1.0 and precision was 0.96 indicating that the method for data extraction was accurate and reliable. For WBC data, recall was 0.76 and precision was 1.0. See Supplementary Figs. 1 and 2 for sample selection methods for analysis of CRP and WBC respectively.

### 3.2 Prevalence of low-grade inflammation (Serum CRP level >3 mg/L)

**Fig. 1:**
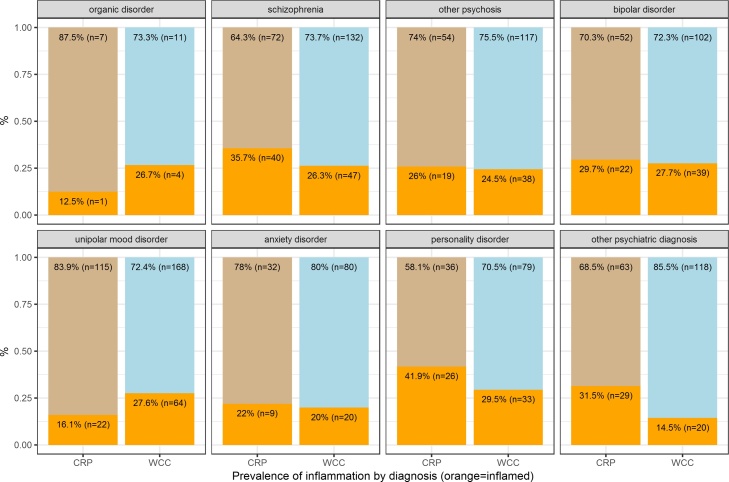
Prevalence of Inflammation (CRP >3 mg/L or WBC >9.4 × 10^9^/L) by Diagnosis. Legend: CRP: proportion of inflamed patients as measured by CRP >3mg/L; WBC: proportion of inflamed patients as measured by WBC >9.4 × 10^9^/L.

**Fig. 2:**
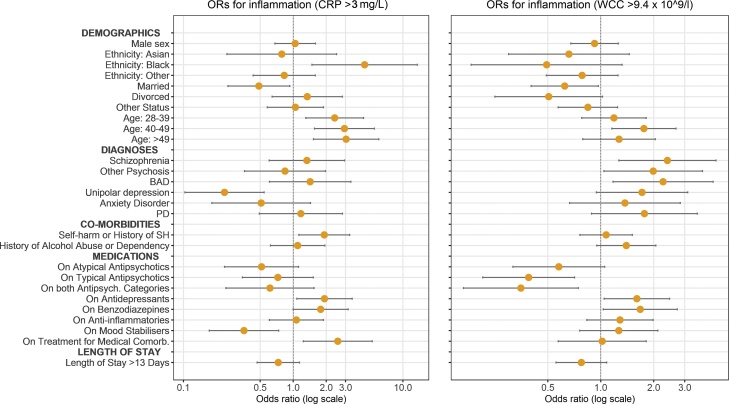
Adjusted Odds Ratios for Inflammation (CRP >3 mg/L or WBC >9.4 × 10^9^/L) for Demographic and Clinical Factors. Legend: BAD: bipolar affective disorder; PD: personality disorder; SH: self-harm. NOTE: Reference categories are: *female* for sex, *white* for ethnicity, *single* for marital status, *18–27* for age, *other* for diagnosis, the *absence* of the condition for each comorbidity, and *≤13 days* for length of stay. ORs are adjusted in a logistic regression model with inflammation (CRP >3 mg/L) as the dependent variable, and sex, age, marital status, ethnicity, main diagnosis, comorbidities, current medication, length of stay as predictor variables.

This analysis included 599 admissions; 48% men, mean age 39 years (SD 13). The prevalence of low-grade inflammation, as defined by serum CRP >3 mg/L, in this sample of acutely unwell, psychiatric inpatients was 28% (see Table 1). The prevalence of inflammation in the major ICD-10 diagnostic groups of psychotic disorders (F20–29), mood disorders (F30–39), neurotic disorders (F40–48) and personality disorders (F60–69) was 32%, 21%, 22% and 42%, respectively (see Fig. 1 and Table 3). In multivariable analyses, a diagnosis of unipolar depression was associated with a decreased risk of inflammation after adjusting for sex, age, marital status, ethnicity, main diagnosis, comorbidities, current medication, and length of admission (adjusted OR = 0.25; 95% CI, 0.11–0.57; p = 0.001). The other diagnoses did not show any significant association with inflammation (see Fig. 2 and Table 2).

**Table 1:** Baseline Characteristics of Sample for CRP analyses.

Domain	Characteristic	Total Sample	Non-Inflamed (CRP ≤3 mg/L)	Inflamed (CRP >3 mg/L)	Test statistic and p value^a^
	Sample size	599	431 (72%)	168 (28%)	
Socio-demographic factors	Age, mean (SD) years	39 (13)	37.5 (13)	42 (12.2)	t = 4.1, df = 324, p < 0.001
	Male sex, *n* (%)	285 (48%)	199 (46%)	86 (51%)	*χ^2^* = 1.02, df = 1, p = 0.31
	Ethnicity, *n* (%)				*χ^2^* = 10.2, df = 4, p = 0.04
	White British	441 (74%)	312 (72%)	129 (77%)	
	White Other	36 (6%)	31 (7%)	5 (3%)	
	Asian	24 (4%)	19 (5%)	5 (3%)	
	Black	17 (3%)	8 (2%)	9 (5%)	
	Other	81 (13%)	61 (14%)	20 (12%)	
	Marital status, *n* (%)				*χ^2^* = 9.5, df = 4, p = 0.05
	Single	316 (53%)	230 (53%)	86 (51%)	
	Married	112 (19%)	87 (20%)	25 (15%)	
	Divorced	48 (8%)	26 (6%)	22 (13%)	
	Other	123 (21%)	88 (20%)	35 (21%)	

Substance use	Past/current self-harm, *n* (%)	135 (22.5%)	88 (20%)	47 (28%)	*χ^2^* = 3.5, df = 1, p = 0.06
	Alcohol misuse, *n* (%)	118 (20%)	78 (18%)	40 (24%)	*χ^2^* = 2.14, df = 1, p = 0.14

Current medication	Antipsychotic prescription, *n* (%)				*χ^2^* = 3, df = 3, p = 0.4
	None	218 (36%)	161 (37%)	57 (34%)	
	Atypical	188 (31%)	137 (32%)	51 (30%)	
	Typical	129 (21.5%)	94 (22%)	37 (22%)	
	Both typical and atypical	62 (10%)	39 (9%)	23 (14%)	
	Antidepressant prescription	216 (36%)	152 (35%)	64 (38%)	*χ^2^* = 0.3, df = 1, p = 0.6
	Benzodiazepine prescription	266 (44%)	184 (43%)	82 (49%)	*χ^2^* = 1.6, df = 1, p = 0.2
	Mood stabiliser prescription	73 (12%)	56 (13%)	17 (10%)	*χ^2^* = 0.7, df = 1, p = 0.4
	Anti-inflammatory prescription^b^	125 (21%)	89 (21%)	36 (21%)	*χ^2^* = 0.01, df = 1, p = 0.9
	Prescriptions for medical co-morbidity	53 (9%)	31 (7%)	22 (13%)	*χ^2^* = 4.5, df = 1, p = 0.03
	Length of admission, mean (SD) days	42 (68)	47 (73)	30 (51)	t = −3, df = 403, p = 0.002

a A *t* test was used to compared mean values between groups (age, length of admission); a chi-squared test was used for categorical variables.

b NSAIDs and opiates.

**Table 2:** Adjusted Odds Ratios for Factors Associated with Low-Grade Inflammation in Acutely Unwell Psychiatric Inpatients on Admission (CRP >3 mg/L).

Characteristic	Groups	OR for inflammation – CRP >3 mg/L (95% CI)	Adjusted^a^ OR for inflammation – CRP >3 mg/L (95% CI)
Sex	Female	1.0 (reference)	1.0 (reference)
	Male	1.22 (0.9–1.7)	1.06 (0.7–1.6)

Age	<28	1.0 (reference)	1.0 (reference)
	28–39	2.15 (1.3–3.7)	2.36 (1.3–4.3)
	40–49	2.17 (1.3–3.7)	2.86 (1.5–5.3)
	>49	2.65 (1.6–4.5)	3.01 (1.5–6.0)

Ethnicity	White	1.0 (reference)	1.0 (reference)
	Asian	0.69 (0.2–1.8)	0.75 (0.2–2.4)
	Black	2.87 (1.1–7.9)	4.21 (1.4–12.8)
	Other	0.84 (0.5–1.4)	0.79 (0.4–1.5)

Marital status	Single	1.0 (reference)	1.0 (reference)
	Married	0.77 (0.5–1.3)	0.49 (0.3–0.95)
	Divorced	2.26 (1.2–4.2)	1.33 (0.6–2.8)
	Other	1.07 (0.7–1.7)	1.07 (0.6–1.9)

Diagnosis	Other (including organic brain disorders)	1.0 (reference)	1.0 (reference)
	Schizophrenia	1.29 (0.7–2.3)	1.37 (0.6–3.0)
	Other psychotic disorder	0.82 (0.4–1.6)	0.86 (0.4–2.0)
	Bipolar mood disorder	0.99 (0.5–1.9)	1.44 (0.6–3.4)
	Unipolar depression	0.45 (0.2–0.8)	0.25 (0.1–0.6)
	Anxiety disorders	0.66 (0.3–1.5)	0.52 (0.2–1.5)
	Personality disorders	1.68 (0.9–3.3)	1.21 (0.5–2.9)

Comorbidity	Self-harm or history of self-harm	1.51 (1.0–2.3)	1.91 (1.1–3.2)
	Personal history of alcohol abuse or dependency	1.42 (0.9–2.2)	1.10 (0.6–1.9)

Medication	On atypical antipsychotics	1.05 (0.7–1.6)	0.52 (0.2–1.1)
	On typical antipsychotics	1.11 (0.7–1.8)	0.71 (0.3–1.5)
	On both typical and atypical antipsychotics	1.67 (0.9–3.0)	0.60 (0.2–1.5)
	On antidepressants	1.13 (0.8–1.6)	1.85 (1.03–3.3)
	On benzodiazepines	1.28 (0.9–1.8)	1.81 (1.01–3.2)
	On NSAIDs and opiates	1.05 (0.7–1.6)	1.09 (0.6–1.9)
	On mood stabilisers	0.76 (0.4–1.3)	0.35 (0.2–0.7)
	On treatment for medical comorbidities	1.95 (1.1–3.5)	2.48 (1.2–5.1)
	Length of stay in hospital above median (>13 days)	0.74 (0.5–1.1)	0.73 (0.5–1.1)

a OR adjusted in a logistic regression model with inflammation (CRP >3 mg/L) as the dependent variable, and sex, age, marital status, ethnicity, main diagnosis, comorbidities, current medication, and length of stay as predictor variables.

**Table 3:** Number and Characteristics of Psychiatric Inpatients by Diagnosis.

Diagnosis	Total *n*	Age, Mean (SD) Years	Male Sex, *n* (%)	White British ethnicity, *n* (%)	*N* with CRP data	Inflammation (CRP >3 mg/L), *n* (%)
F00-F09 Organic Mental Disorders	17	52 (12)	8 (47%)	13 (77%)	8	1 (13%)
F20-F29 Psychotic Disorders	445	40 (12)	284 (64%)	275 (62%)	185	59 (32%)
F20 Schizophrenia	232	40 (11)	165 (71%)	150 (65%)	112	40 (36%)
F21-29 Other Psychoses	213	40 (13)	119 (56%)	125 (59%)	73	19 (26%)
F30-F39 Mood Disorders	523	41 (13)	245 (47%)	381 (73%)	211	44 (21%)
F30-31 Bipolar	191	44 (13)	87 (46%)	134 (70%)	74	22 (30%)
F32-39 Unipolar or Unspecified	332	40 (13)	158 (48%)	247 (74%)	137	22 (16%)
F40-F48 Neurotic, stress- related and somatoform dis.	131	34 (11)	69 (53%)	102 (78%)	41	9 (22%)
F60-F69 Personality Disorders	149	33 (10)	51 (34%)	121 (81%)	62	26 (42%)
Other F diagnoses	188	39 (13)	122 (65%)	139 (74%)	92	29 (32%)

#### 3.2.1 Association with sociodemographic factors

Older age, black ethnicity and being married were associated with inflammation, after adjusting for sex, age, marital status, ethnicity, main diagnosis, comorbidities, current medication, and length of admission. Sex was not associated with inflammation (see Fig. 2 and Table 1, Table 2).

#### 3.2.2 Association with prescribed medication

Low-grade inflammation was associated with current treatments with antidepressants (adjusted OR = 1.85; 95% CI:1.03–3.33; p = 0.038), benzodiazepines and/or hypnotics (adjusted OR = 1.81; 95% CI:1.01–3.22; p = 0.045). Mood stabilisers were associated with a decreased risk of inflammation (adjusted OR = 0.35; 95% CI:0.17–0.73; p = 0.005). Non-steroidal anti-inflammatory drugs and painkillers were not associated with inflammation (see Table 2 and Fig. 2). There was no association between the number of prescribed psychotropic medications and the risk of inflammation. Inflammation was not associated with antipsychotic medications after controlling for potential confounders.

#### 3.2.3 Association with medical comorbidity

Patients with medical comorbidities were more likely to be inflamed (adjusted OR = 2.48; 95% CI:1.20–5.13; p = 0.01), after correcting for sex, age, marital status, ethnicity, main diagnosis, other comorbidities, current medication, and length of admission (see Table 2 and Fig. 2).

#### 3.2.4 Association with self-harm, drug and alcohol use

Patients with a current or historical diagnosis of self-harm or poisoning were more likely to be inflamed compared with those without such history (adjusted OR = 1.91; 95% CI:1.12–3.25; p = 0.02). History of alcohol abuse or alcohol dependency were not associated with inflammation (See Table 2 and Fig. 2).

#### 3.2.5 Association with length of admission

Low-grade inflammation was not associated with total length of admission in analyses using length of admission as a binary variable (See Fig. 2).

#### 3.2.6 Sensitivity analyses excluding admissions with CRP >20 mg/L

In sensitivity analyses we excluded admissions of patients who presented CRP levels >20 mg/L. The results remained mostly unchanged (see Supplementary Fig. 3), except that the associations between inflammation and marital status and black ethnicity were no longer statistically significant.

### 3.3 Results for additional analyses using high white cell count (>9.4 × 10^9^/L) to define inflammation

#### 3.3.1 Relationship between CRP and WBC

Data on both CRP and WBC were available for 325 participants. Logistic regression using CRP as a binary dependent variable (CRP >3 mg/L vs ≤3 mg/L) and WBC as a continuous predictor variable showed that CRP was associated with WBC (beta = 0.13, SE = 0.04, z = 3.068, p = 0.002). The OR for high CRP (>3 mg/L) for those with high WBC (>9.4 × 10^9^/L) was 2.27 (95% CI, 1.32–3.90, p = 0.003).

#### 3.3.2 Prevalence of inflammation using WBC (>9.4 × 10^9^/L)

This analysis included 1072 admissions; 56% men, mean age 39 years (SD 13). The prevalence of low-grade inflammation, defined as WBC >9.4 × 10^9^/L, in this sample was 25% (see Supplementary Table 2). Table 4 provides a summary of the significant findings, comparing them to those obtained using CRP.

**Table 4:** Summary of significant findings.

	OR for inflammation defined as CRP >3 mg/L	OR for inflammation defined as WBC >9.4*10^9^/L	Concordance between CRP and WBC analyses
Black ethnicity	↑	⇣	N
Married status	↓	↓	↓
Older age	↑	↑	↑
Diagnosis of schizophrenia	⇡	↑	⇡
Diagnosis of bipolar disorder	⇡	↑	⇡
Diagnosis of unipolar depression	↓	⇡	N
Current or past self-harm	↑	↔	N
Current treatment with typical or atypical antipsychotics	⇣	↓	⇣
Current treatment with antidepressants	↑	↑	↑
Current treatment with benzodiazepines	↑	↑	↑
Current treatment with mood stabilisers	↓	⇡	N
Current treatment for medical comorbidity	↑	↔	N

Legend: ↑ = OR >1 and statistically significant suggesting these factors increase inflammation; ↓ = OR <1 and statistically significant suggesting these factors decrease inflammation; ⇡ = OR >1 but not statistically significant suggesting these factors may decrease inflammation; ⇣ = OR <1 but not statistically significant suggesting these factors may decrease inflammation; ↔ = OR not statistically different from 1; N = results are not concordant between CRP and WBC.

#### 3.3.3 Association of high WBC (>9.4 × 10^9^/L) with demographic factors

High WBC (>9.4 × 10^9^/L) was associated with married status (adjusted OR = 0.62; 95% CI:0.4–0.97; p = 0.04), older age (adjusted OR for age 40–49, compared with age <28 = 1.75; 95% CI:1.1–2.7; p = 0.01), but not with ethnicity (see Supplementary Tables 2, 3 and Fig. 2).

#### 3.3.4 Association of high WBC (>9.4 × 10^9^/L) with clinical factors

High WBC (>9.4 × 10^9^/L) was associated with a diagnosis of schizophrenia (adjusted OR = 2.41; 95% CI:1.3–4.5; p < 0.01), other psychotic disorders (adjusted OR = 1.99; CI:1.04–3.8; p = 0.04), and bipolar affective disorder (adjusted OR = 2.26; 95% CI:1.2–4.4; p = 0.01), after adjusting the model for age, sex, ethnicity, marital status, main diagnosis, self-harm, alcohol abuse, medical comorbidities, current medications, length of admission. Current treatments with typical antipsychotics (adjusted OR = 0.39; 95% CI:0.2–0.7; p = 0.002), and typical plus atypical antipsychotics (adjusted OR = 0.35; 95% CI:0.2–0.7; p = 0.006) were associated with lower WBC. On the other hand, current treatment with antidepressants (adjusted OR = 1.60; 95% CI:1.04–2.4; p = 0.03) and benzodiazepines (adjusted OR = 1.68; 95% CI:1.04–2.7; p = 0.04) were associated with high WBC (see Fig. 2 and Supplementary Table 3).

## 4 Discussion

We studied low-grade inflammation in acute psychiatric inpatients on admission across different diagnostic groups. Overall, over a quarter of all patients in our sample showed evidence of inflammation. Evidence of low-grade inflammation was present in all major diagnostic groups, with prevalences ranging from 12 to 40% depending on the measure. A number of sociodemographic and clinical factors were associated with inflammation. Older age and current treatment with antidepressants and benzodiazepines were associated with an increased risk of inflammation after controlling for potential confounders. These findings were consistent across analyses using CRP and WBC as markers of inflammation. Being married appeared to be protective against inflammation, but evidence for this association did not persist after excluding patients with CRP >20 mg/L. There was some evidence that inflammation was associated with current/past self-harm and with being on treatment for medical comorbidities such as diabetes, hypertension and dyslipidaemia. Diagnoses of schizophrenia, other psychotic disorders, and bipolar disorder were associated with an increased risk of inflammation, while treatments with mood stabilisers or antipsychotics were associated decreased risk of inflammation.

There could be many reasons for a high prevalence of low-grade inflammation in acutely unwell psychiatric patients. Psychological stress can activate the immune system (Padgett and Glaser, 2003). Exposure to early-life adversity, common in psychiatric patients, can increase levels of inflammation in adulthood (Baumeister et al., 2016). Inflammation could be a marker of co-morbid inflammatory physical illness. However, accumulating evidence suggests that inflammation could be an intrinsic part of psychiatric illnesses. Meta-analyses of cross-sectional studies show increased levels of inflammatory markers in acutely unwell patients with depression and psychosis (Howren et al., 2009; Potvin et al., 2008). Population-based longitudinal studies have reported that higher levels of IL-6 and CRP are associated with symptoms/diagnosis of depression, mania and psychosis subsequently in life (Gimeno et al., 2009; Hayes et al., 2017; Khandaker et al., 2014; Khandaker et al., 2017; Metcalf et al., 2017; Zalli et al., 2016), suggesting low-grade inflammation could be a cause for these illnesses, rather than simply being a consequence.

Our results are in line with previous studies reporting prevalence of low-grade inflammation (CRP >3 mg/L) in depression (Cizza et al., 2009; Raison et al., 2013; Wium-Andersen et al., 2013). These studies have investigated inflammation in depression in specific contexts such as in premenopausal women, in depressed outpatients, or in the general population. Our study adds to previous findings by reporting the prevalence of inflammation a) in a psychiatric inpatient population, and b) in other patient groups.

A previous study examined the prevalence of inflammation in the general population, and found that, using reference limits set at the time of the analysis, for WBC 21.4% of the population had an above-reference value, and for CRP 22.2% of the population had above-reference results (Andersen et al., 2014). However, it should be noted that cut-offs for WBC/CRP levels used to define low-grade inflammation in our study were different, as we were interested in low-grade inflammation.

Associations of inflammation with older age, marital status and ethnic minority status are consistent with previous studies (Khera et al., 2005; Sbarra, 2009; Wener et al., 2000; Woloshin and Schwartz, 2005), although we have not seen any association with sex. In our sample, current treatments with antidepressants, anxiolytics/hypnotics were robustly associated with inflammation. This is consistent with previous studies reporting elevated levels of inflammatory markers in patients with depression (Dowlati et al., 2010; Goldsmith et al., 2016; Haapakoski et al., 2015; Howren et al., 2009). Furthermore, raised levels of the inflammatory cytokine IL-6 in childhood are associated with an increased risk of developing depression and psychosis in young adulthood (Khandaker et al., 2014), and persistent depressive symptoms during the second decade of life (Khandaker et al., 2017). However, a diagnosis of depression was associated with lower risk of inflammation, which is surprising. It is possible that antidepressant prescription is a better proxy for current depression in our sample; data on diagnoses obtained from electronic health records were historical, while prescription data refers to the current admission. Nevertheless, there was some evidence that high WBC was associated with a diagnosis of depression although this was not statistically significant. We did not have repeat measures of CRP/WBC at the end of the admission, so it was not possible to examine the association of inflammation with treatment response.

Association of inflammation with self-harm and medical comorbidities are consistent with previous studies. Previous studies have reported an association between inflammation and suicidal ideation/behaviour (Gibbs et al., 2016; Park and Kim, 2017). Self-harm and suicidal ideas are markers of psychiatric multi-morbidity (Hui et al., 2013), so reflect patients with greater psychological distress. Previous studies have reported that inflammatory markers are associated with the severity of depressive symptoms (Köhler-Forsberg et al., 2017) and with persistent depressive symptoms (Khandaker et al., 2017; Zalli et al., 2016). Both cardiovascular disease and diabetes mellitus are associated with low-grade inflammation (Koenig et al., 1999; Pearson et al., 2003). There is evidence of insufficient glucocorticoid signalling and elevated inflammation in coronary heart disease patients with comorbid depression (Nikkheslat et al., 2015). However, data on medical comorbidities were often missing in the electronic health record, so prescribed medications were used as a proxy.

A decreased risk of inflammation in patients taking mood stabilisers is consistent with known anti-inflammatory effects of lithium (Kucharz et al., 1993; Sluzewska et al., 1997) and valproate (Yuen et al., 2010). CRP levels increase during a manic phase, which is at least partially reversed by treatment with mood stabilisers (Fernandes et al., 2016). Our finding is also consistent with a previous longitudinal study reporting an association between childhood IL-6 levels and lifetime hypomanic symptoms assessed in adulthood (Hayes et al., 2017). Interestingly, inflammation was not associated with the prescription of NSAIDs, which was used as a proxy for chronic inflammatory illness. This suggests that the inflammation seen in psychiatric patients in our sample might not be driven solely by co-morbid inflammatory medical conditions, but it could rather be inherent to their psychiatric illness.

The diagnoses of schizophrenia and bipolar affective disorder were also associated with an increased risk of inflammation measured through WBC, while antipsychotic treatment was associated with a protective effect. The results are consistent with recent evidence showing there is an increase in peripheral inflammatory markers in schizophrenia, which normalised with antipsychotic treatment. IL-1β, IL-6, and transforming growth factor-β (TGF-β) are schizophrenia state markers, as they increase in acute relapses and first episode psychosis and normalize with antipsychotic treatment (Miller et al., 2011). In contrast, IL-12, IFN-γ, TNF-α, and soluble IL-2 receptor are trait markers, as levels remain elevated in acute exacerbations and following antipsychotic treatment. Furthermore, IL-6 levels correlate positively with symptom severity (Miller et al., 2011). There is evidence that elevated CRP in adolescence is associated with increased risk of psychosis later on in life (Metcalf et al., 2017).

The limitations of this study need to be considered carefully. This study is based on retrospective analysis of data from an electronic health record that was not created for the purpose of research. Although the work demonstrates that routine clinical databases can be used to address important research questions, there are limitations to using routine clinical data. Missing data is a key issue. Although all patients admitted to CPFT inpatient hospitals are offered a physical examination including a blood test (which includes WBC and CRP routinely), only a subset of all potentially eligible patients had data on inflammatory markers. Approximately 41% of the admissions that we considered (Supplementary Figs. 1 and 2) had recorded blood results within 14 days of admission. The remaining 59% who did not have blood results recorded within 2 weeks of admission were not included in our study; these patients might have refused venepuncture at the time of admission or had bloods taken after 14 days of coming into hospital, which we excluded from our study to minimize potential effects of hospital treatment. CRP and WBC data recall and precision also affected the probability of inclusion into the analytic sample, as described in the methods. While the possibility of selection bias due to missing data cannot be ruled out, it is unlikely that blood tests were offered primarily because of a suspected physical illness. Therefore, the increased prevalence of inflammation in psychiatric patients is unlikely to be due to a physical illness. Data on diagnosis were also missing for many patients. We included only patients with a clinician-coded ICD-10 diagnosis to increase accuracy. Therefore, diagnosis data are more specific than sensitive. However, patients often more than one psychiatric diagnosis, so we used a hierarchical approach to assign a single main diagnosis to each patient. This hierarchical approach has been used previously in psychiatric research (Foulds and Bedford, 1975). It is possible that for some patients the reason for admission was different from their main diagnosis, leading to misclassification of diagnosis. However, this is unlikely to be an issue for a majority of patients.

To explore potential selection bias due to missing data, we compared demographic factors between the analytic sample and missing sample (all unique patients meeting all selection criteria but with no recorded CRP on admission). These samples were similar in terms of sociodemographic factors except for marital status, suggesting that, while the possibility of selection bias cannot be ruled out, it is unlikely to be a major issue (see Supplementary Table 1). However, the prevalence of inflammation in patients with depression observed in our sample is comparable to previous studies of depression (Cizza et al., 2009; Raison et al., 2013; Wium-Andersen et al., 2013): this is reassuring. The database would not differentiate between first and subsequent admissions for a specific patient for a given diagnosis, therefore we were unable to compare recent onset cases with those with chronic illness.

A recent clinical study found associations between WBC and greater bipolar severity (Köhler et al., 2017). We did not have data on severity of illness. However, the fact that patients were admitted to hospital is an indication that they were severely unwell.

Our study is limited to patients who were admitted to hospital. To our knowledge, this is one of the first studies to examine the prevalence of inflammation in psychiatric inpatients from all major ICD-10 diagnostic groups. However, the decision to admit is often guided by clinical risk perceived by clinicians, so the findings may not be generalizable to all patients. We did not have readily available data on BMI, smoking or recreational drug use. Therefore, the association between inflammation and diagnosis of psychosis might reflect antipsychotic induced obesity/metabolic disturbance. Due to the lack of an electronic prescribing system, we used a natural language processing software to extract names of prescribed drugs from medical notes. This system has been previously validated in a similar study (Cardinal et al., 2015). However, while we were able to ascertain whether a particular drug was prescribed or not, we could not ascertain specific indication for that prescription. The same psychotropic drugs can be prescribed for many disorders. Therefore, the relationship between prescribed antidepressant and inflammation might reflect that inflammation is associated with a range of disorders for which these drugs are prescribed, rather than depression specifically.

## 5 Conclusions

In summary, our findings indicate that a large minority of acutely unwell psychiatric patients show evidence of low-grade systemic inflammation, regardless of their diagnosis. Low-grade inflammation is associated with a number of socio-demographic and clinical factors, which may help to characterise an inflammatory sub-type of the major psychiatric disorders. For conditions such as depression, it is known that an inflammatory phenotype is associated with treatment resistance. This is not known for all other psychiatric disorders. Characterising the inflammatory sub-type of psychiatric disorders could therefore allow to predict which patients might be treatment resistant, and incentivises work into elucidating the clinical phenotype of inflamed patients presenting with other psychiatric conditions.

However, before our work can be generalised further work based on other settings (e.g., outpatients) and samples (e.g., general population) is required.

## Conflicts of interest

The authors have no conflict of interests to declare.
