# Supplementary material for: Prevalence and correlates of low-grade systemic inflammation in adult psychiatric inpatients: An electronic health record-based study
Source: Psychoneuroendocrinology. 2018 May;91:226–34. doi: 10.1016/j.psyneuen.2018.02.031 (PMC5910056; doi:10.1016/j.psyneuen.2018.02.031)
Supplement: Supplementary file 1 [file mmc1.docx]

# SUPPLEMENTARY INFORMATION

SUPPLEMENTARY INFORMATION 1

SUPPLEMENTARY METHODS 2

Setting 2

Coding, Recall and Precision of CRP and WBC Data 2

Coding of Diagnosis Data 2

Coding of Prescribed Medications 2

Length of admission 3

SUPPLEMENTARY TABLES 4

Supplementary Table 1: Comparison of Demographic Characteristics Between Patients with or without Available CRP Blood Results 4

Supplementary Table 2: Baseline Characteristics of Sample for WBC analyses 5

Supplementary Table 3: Odds Ratios for Inflammation on admission (WBC >9.4 x10^9^/l) 7

Supplementary Table 4: Classes and Names of Prescribed Medications Included in Analysis 9

SUPPLEMENTARY FIGURES 10

Supplementary Figure 1: flow diagram of sample selection – data for CRP 10

Supplementary Figure 2: flow diagram of sample selection – data for WBC 11

Supplementary Figure 3: Results for Sensitivity Analysis: Adjusted Odds Ratios and 95% CI for Inflammation (CRP >3mg/L) for Demographic and Clinical Factors, after the exclusion of admissions with CRP >20 mg/L 12

Supplementary references 13

## SUPPLEMENTARY METHODS

### Setting

CPFT provides secondary mental, social and physical health care to a population of approximately 800,000 across Cambridgeshire and Peterborough, two geographically defined areas in England (Cambridgeshire County Council Research and Performance Team, 2013). Psychiatric patients are admitted to one of two hospitals, one in Cambridge and one in Peterborough.

### Coding, Recall and Precision of CRP and WBC Data

Our sample size was limited partly by the availability of CRP and WBC data. Blood samples from different areas of the Trust were sent to different laboratories; some of these labs used low-sensitivity assays that could only detect CRP levels >10mg/L. We had to exclude this data set as we were interested in sub-clinical levels of inflammation that had previously linked with an increased risk of heart disease (Pearson et al., 2003; Ridker, 2003). For WBC data we chose a cut-off equivalent to the 75^th^ percentile of the distribution of WBC values in our sample. This is in line with our CRP data, where 4mg/L represented the 72^nd^ percentile. We did not choose the upper limit of the WBC reference range as we were not interested in selecting patients with active infection (pathologically high WBC), while we wanted to select a subsample of healthy people with low-grade inflammation.

Recall was defined as the probability of retrieving a record, given that it was relevant. For example, recall for patients having a given CRP level, e.g. CRP = 4, would be P(electronically identified as having CRP=4 | actually having a recorded CRP level of 4 mg/L).

Precision was defined as the probability of a record being relevant, given that it was retrieved. For example, precision for the same search would be P(actually having a recorded CRP level of 4 mg/L | electronically identified as having CRP=4).

Recall and precision were assessed for CRP and WBC levels by manually checking the correspondence of 100 random records with the automatically extracted values we had obtained.

### Coding of Diagnosis Data

All psychiatric diagnoses were analysed as they appear in ICD-10, except for the following:

- Self-harm or poisoning included ICD-10 diagnoses of “Intentional self-harm” (X60-X84), “Personal history of self-harm” (Z91.5), “Poisoning by narcotics and psychodysleptics” (T40), “Poisoning by diuretics and other and unspecified drugs, medicaments and biological substances” (T50)
- Alcohol misuse included ICD-10 diagnoses of “Mental and behavioural disorders due to use of alcohol, Harmful use” (F10.1), “Dependence syndrome” (F10.2) and “Unspecified mental and behavioural disorder”, (F10.9)

### Coding of Prescribed Medications

Medical notes were searched for free text using natural language processing software developed to extract drug names and common misspellings using the General Architecture for Text Engineering (GATE) (Cunningham et al., 2013), to obtain automatically generated drug histories. All medications with more than 2 occurrences were considered. Supplementary Table 3 summarizes the coding of medication.

Medication information was only used if it contained dose information and was written in the present tense indicating current use.

### Length of admission

Length of admission (in days) was extracted from health records. In addition to treating as a continuous variable, we created a binary variable using median (>13 days vs <=13 days).

## SUPPLEMENTARY TABLES

### Supplementary Table 1: Comparison of Demographic Characteristics Between Patients with or without Available CRP Blood Results

|  | **Analytic Sample**  Unique patients with recorded admission CRP results (and other selection criteria) | **Missing Sample**  Unique patients meeting all selection criteria but no recorded CRP on admission | **Test statistic and p value^1^** |
| --- | --- | --- | --- |
| **Sample size** | N=1493 | N=1768 |  |
| **Age**  mean (SD) | 39.1 (13.0) | 38.24 (12.28) | t-test: t = -1.9,  df = 3104.8,  p = 0.06 |
| **Male sex**  N (%) | 775 (52%) | 956 (54%) | *χ^2^*= 1.52, df = 1, p=0.22 |
| **Ethinicity,** N (%)  White  Black  Asian  Other | 1158 (77.5%)  41 (3%)  55 (4%)  239 (16%) | 1336 (76%)  39 (2%)  60 (3%)  333 (19%) | *χ^2^*= 5.3, df = 3, p=0.15 |
| **Marital status,** N (%)  Single  Married  Divorced  Widowed | 698 (66%)  256 (24%)  92 (9%)  12 (1%) | 753 (61%)  380 (31%)  94 (8%)  10 (1%) | *χ^2^*= 12.6, df = 3, p= 0.006 |

^1^ A *t* test was used to compared mean values between groups (age); a chi-squared test was used for categorical variables.

### Supplementary Table 2: Baseline Characteristics of Sample for WBC analyses

| **Domain** | **Characteristic** | **Total Sample** | **Non-Inflamed (WBC <=9.4 x10^9^/l)** | **Inflamed (WBC >9.4 x10^9^)** | **Test statistic and p value^1^** |
| --- | --- | --- | --- | --- | --- |
|  | All | 1072 | 807 (75%) | 265 (25%) |  |
| Demographics | Age, mean (SD) years | 39 (13) | 39 (13) | 40 (12) | t-test: t=-0.95, df=487, p<0.34 |
|  | Male sex, *n* (%) | 600 (56%) | 455 (56%) | 145 (55%) | *χ^2^*=0.16, df=1, p=0.69 |
|  | Ethnicity, *n* (%)  White British  Asian  Black  Other | 761 (71%)  51 (5%)  36 (3%)  224 (21%) | 560 (69%)  42 (5%)  31 (4%)  174 (22%) | 201 (76%)  9 (3%)  5 (2%)  50 (19%) | *χ^2^*=5.5, df=3, p=0.14 |
|  | Marital Status, *n* (%)  Single  Married  Divorced  Widowed  Other | 520 (49%)  195 (18%)  67 (6%)  11 (1%)  279 (26%) | 374 (46%)  154 (19%)  55 (7%)  8 (1%)  216 (27%) | 146 (55%)  41 (15%)  12 (5%)  3 (1%)  63 (24%) | *χ^2^*: X=7.0, df=4, p=0.13 |
| Drugs and alcohol | Past/current self-harm, *n* (%) | 352 (33%) | 245 (30%) | 97 (37%) | *χ^2^*=3.3, df=1, p=0.07 |
|  | Alcohol misuse | 232 (22%) | 168 (21%) | 64 (24%) | *χ^2^*=1.12, df=1, p=0.29 |
| Current Medication | Antipsychotic prescription  None  Atypical  Typical  Both typical and atypical | 468 (44%)  267 (25%)  215 (20%)  122 (11%) | 357 (44%)  186 (23%)  171 (21%)  93 (12%) | 111 (42%)  81 (31%)  44 (17%)  29 (11%) | *χ^2^*=6.9, df=3, p=0.07 |
|  | Antidepressant prescription | 313 (29%) | 218 (27%) | 95 (36%) | *χ^2^*=7.1, df=1, p=0.007 |
|  | Benzodiazepine prescription | 446 (42%) | 321 (40%) | 125 (47%) | *χ^2^*=4.2, df=1, p=0.04 |
|  | Mood stabiliser prescription | 115 (11%) | 80 (10%) | 35 (13%) | *χ^2^*=1.9, df=1, p=0.2 |
|  | Anti-inflammatory prescription^3^ | 193 (18%) | 138 (17%) | 55 (21%) | *χ^2^*=1.6, df=1, p=0.2 |
|  | Prescriptions for physical co-morbidity^2^ | 81 (8%) | 59 (7%) | 22 (8%) | *χ^2^*=0.2, df=1, p=0.7 |
|  | Length of admission, mean (SD) days | 31 (48) | 31 (50) | 29 (43) | t-test: t=0.6, df=510, p=0.52 |

^1^ A *t* test was used to compared mean values between groups (age, length of stay); a chi-squared test was used for categorical variables.

^2^ The presence of medical co-morbidities was inferred by the presence of prescriptions for anti-hypertensives, diuretics, antidiabetics, statins, anti-aggregants, anti-coagulants or medication for the management of dyslipidaemias.

^3^ NSAIDs and opiates.

### Supplementary Table 3: Odds Ratios for Inflammation on admission (WBC >9.4 x10^9^/l)

The *n*s for the following table can be found in Supplementary Table 1

| **Characteristic** | **Groups** | **OR for inflammation - WBC >9.4 x10^9^/l (95% CI)** | **Adjusted^1^ OR for inflammation - WBC >9.4 x10^9^/l (95% CI)** |
| --- | --- | --- | --- |
| **Sex** | **Female** | 1.0 (reference) | 1.0 (reference) |
|  | **Male** | 1.07 (0.8-1.4) | 0.93 (0.7-1.3) |
| **Age** | **<28** | 1.0 (reference) | 1.0 (reference) |
|  | **28-39** | 1.19 (0.8-1.8) | 1.19 (0.8-1.8) |
|  | **40-49** | 1.62 (1.1-2.4) | 1.75 (1.1-2.7) |
|  | **>49** | 1.08 (0.7-1.6) | 1.27 (0.8-2.0) |
| **Ethnicity** | **White** | 1.0 (reference) | 1.0 (reference) |
|  | **Asian** | 0.61 (0.27-1.21) | 0.66 (0.3-1.5) |
|  | **Black** | 0.46 (0.15-1.10) | 0.49 (0.2-1.3) |
|  | **Other** | 0.80 (0.56-1.13) | 0.88 (0.6-1.3) |
| **Marital status** | **Single** | 1.0 (reference) | 1.0 (reference) |
|  | **Married** | 0.68 (0.5-1.0) | 0.62 (0.4-0.96) |
|  | **Divorced** | 0.56 (0.3-1.1) | 0.51 (0.3-1.0) |
|  | **Other** | 0.75 (0.5-1.0) | 0.82 (0.6-1.2) |
| **Diagnosis** | **Other (including organic brain disorders)** | 1.0 (reference) | 1.0 (reference) |
|  | **Schizophrenia** | 1.91 (1.1-3.3) | 2.41 (1.3-4.5) |
|  | **Other psychotic disorder** | 1.74 (0.99-3.1) | 1.99 (1.04-3.8) |
|  | **Bipolar mood disorder** | 2.05 (1.2-3.7) | 2.26 (1.2-4.4) |
|  | **Unipolar depression** | 2.04 (1.2-3.5) | 1.73 (0.95-3.1) |
|  | **Anxiety disorders** | 1.34 (0.7-2.6) | 1.39 (0.7-2.9) |
|  | **Personality disorder** | 2.23 (1.2-4.1) | 1.77 (0.9-3.5) |
| **Comorbidity** | **Self-harm or history of self-harm** | 1.32 (0.99-1.8) | 1.07 (0.8-1.5) |
|  | **Personal history of alcohol abuse or dependency** | 1.21 (0.9-1.7) | 1.39 (0.9-2.1) |
| **Medication** | **On atypical antipsychotics** | 1.40 (1.0-2.0) | 0.57 (0.3-1.04) |
|  | **On typical antipsychotics** | 0.83 (0.6-1.2) | 0.39 (0.2-0.7) |
|  | **On both typical and atypical antipsychotics** | 1.01 (0.6-1.6) | 0.35 (0.2-0.7) |
|  | **On antidepressants** | 1.51 (1.1-2.0) | 1.60 (1.04-2.4) |
|  | **On benzodiazepines** | 1.35 (1.02-1.8) | 1.68 (1.04-2.7) |
|  | **On NSAIDs and opiates** | 1.27 (0.9-1.8) | 1.28 (0.8-2.0) |
|  | **On mood stabilisers** | 1.39 (0.9-2.1) | 1.27 (0.8-2.1) |
|  | **On treatment for medical comorbidities** | 1.15 (0.7-1.9) | 1.01 (0.6-1.8) |
|  | **Length of stay in hospital above median (> 13 days)** | 0.90 (0.7-1.2) | 0.78 (0.6-1.09) |

^1^ OR adjusted in a logistic regression model with inflammation (>9.4 x10^9^/l) as the dependent variable, and sex, age, marital status, ethnicity, main diagnosis, comorbidities, current medication, and length of stay as predictor variables.

### Supplementary Table 4: Classes and Names of Prescribed Medications Included in Analysis

| **Class** | **Medications in order of frequency** |
| --- | --- |
| Antipsychotics | Olanzapine, quetiapine, haloperidol, aripiprazole, risperidone, clozapine, amisulpride, paliperidone, flupenthixol, zuclopenthixol, chlorpromazine, sulpiride, levomepromazine, pipotiazine, prochlorperazine, pimozide, clopixol, trifluoperazine, asenapine, fluphenazine, pericyazine |
| Antidepressants | Sertraline, mirtazapine, venlafaxine, citalopram, fluoxetine, paroxetine, duloxetine, amitriptyline, escitalopram, trazodone, lofepramine, clomipramine, buspirone, bupropion, fluvoxamine, imipramine, moclobemide, nortriptyline |
| Benzodiazepines and sleep inducers | Zopiclone, lorazepam, diazepam, clonazepam, temazepam, chlordiazepoxide, zolpidem, melatonin, cloralbetaine, chloralhydrate, midazolam, nitrazepam, clobazam |
| Mood stabilisers | Valproate, lithium, lamotrigine, carbamazepine, topiramate |
| Medication for medical comorbidities | Propranolol, simvastatin, metformin, ramipril, amlodipine, atenolol, spironolactone, lisinopril, bisoprolol, furosemide, gliclazide, warfarin, bumetanide, diltiazem, insulin, rivaroxaban, atorvastatin, bendroflumethiazide, clopidogrel, digoxin, exenatide, metoprolol, nifedipine, sitagliptin |
| NSAIDS and pain control medication | Paracetamol, ibuprofen, codeine, morphine, aspirin, naproxen, buprenorphine, co-codamol, oxycodone, oxybutynin, dihydrocodeine, fentanyl, bisacodyl |
| Antibiotics (exclusion criterium) | Amoxicillin, co-amoxiclav, doxycycline, trimethoprim, flucloxacillin, metronidazole, nitrofurantoin, clarithromycin, cefalexin, ciprofloxacin, erythromycin, vancomycin, clotrimazole, fluconazole, hydroxychloroquine, nystatin, aciclovir, ceftriaxone, clindamycin, clotrimazole, lymecycline, minocycline, tetracycline |
| Oral steroids (exclusion criterium) | Prednisolone, tibolone, fludrocortisone |

## SUPPLEMENTARY FIGURES

### Supplementary Figure 1: flow diagram of sample selection – data for CRP


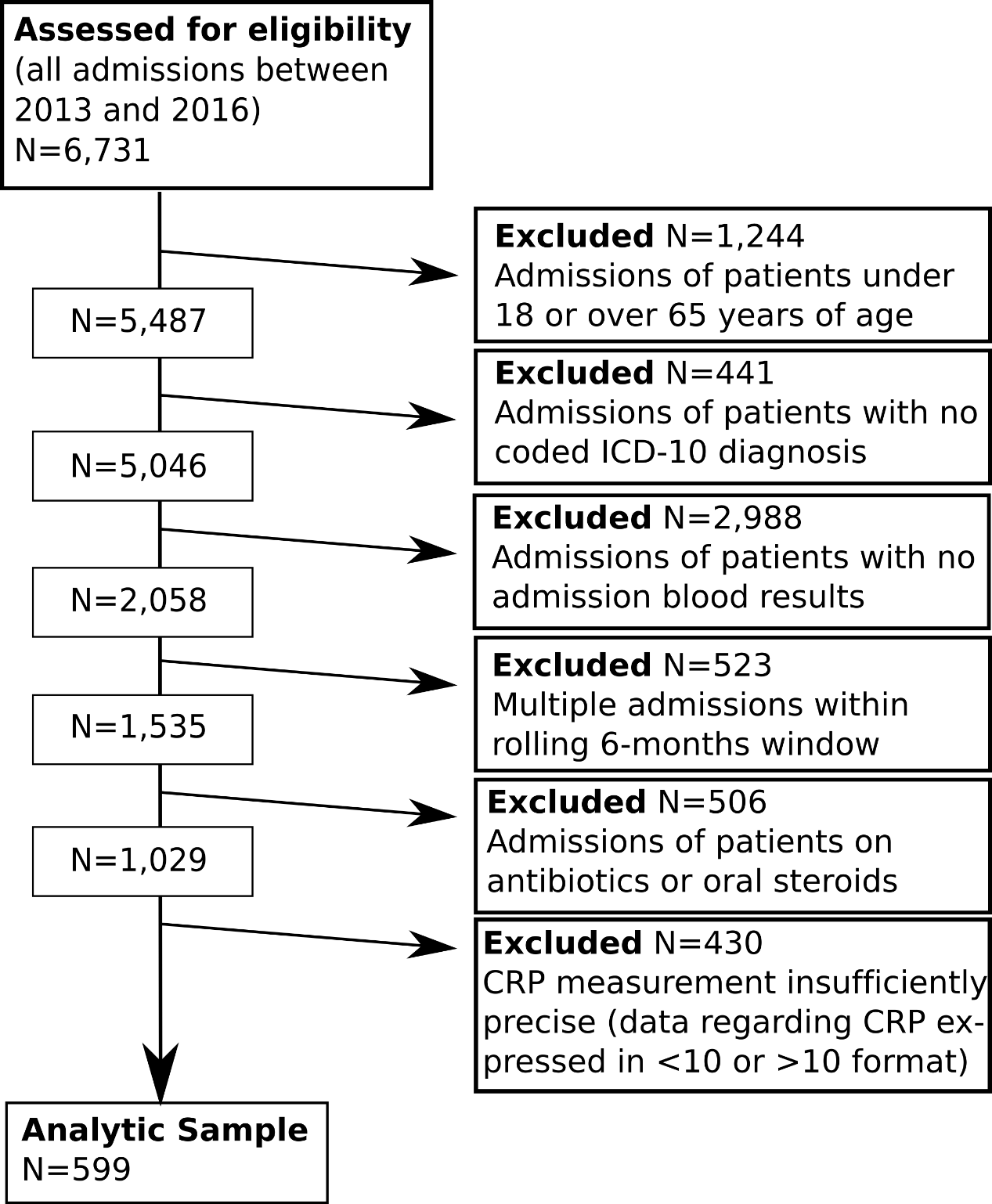


### Supplementary Figure 2: flow diagram of sample selection – data for WBC


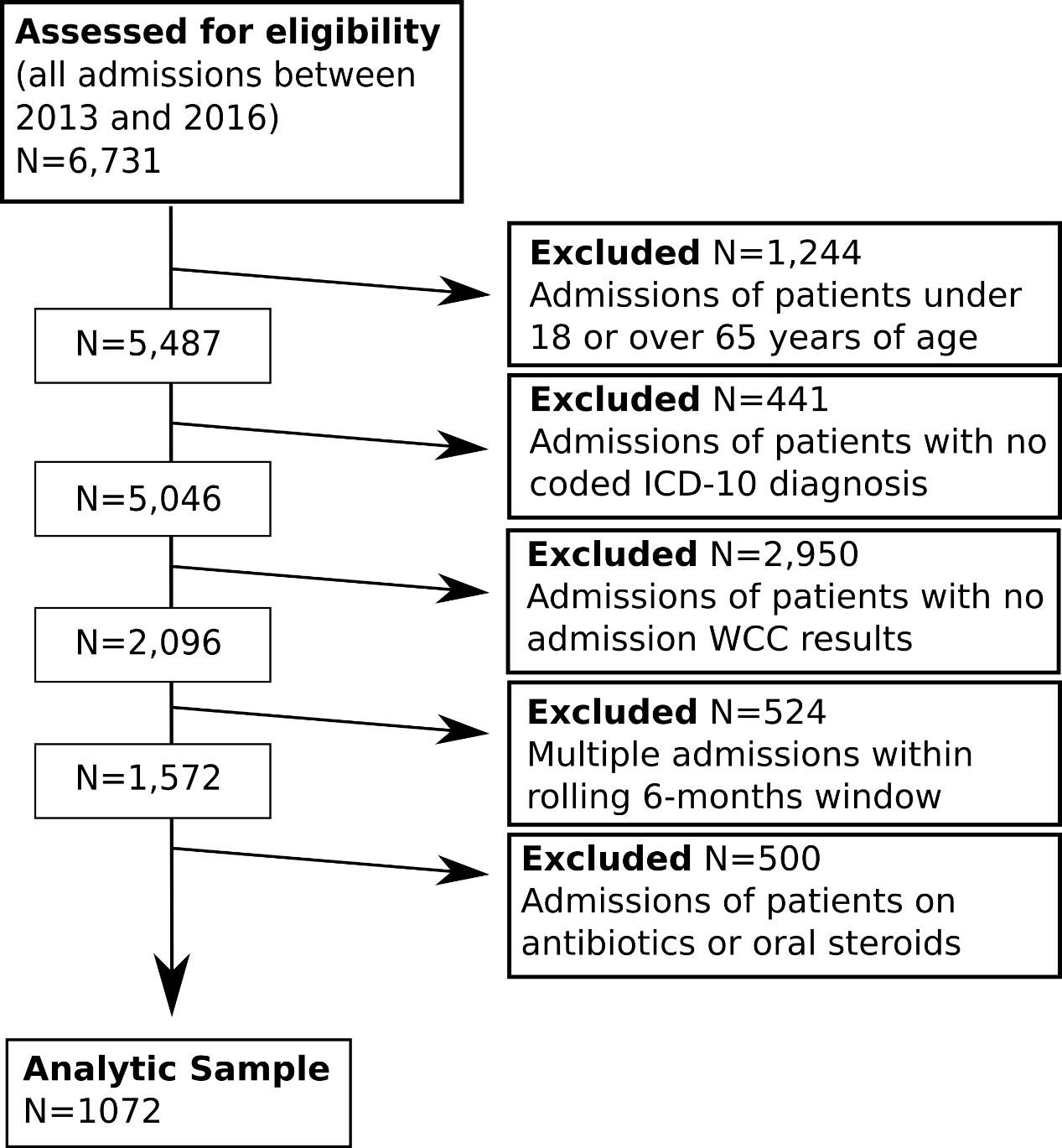


### Supplementary Figure 3: Results for Sensitivity Analysis: Adjusted Odds Ratios and 95% CI for Inflammation (CRP >3mg/L) for Demographic and Clinical Factors, after the exclusion of admissions with CRP >20 mg/L


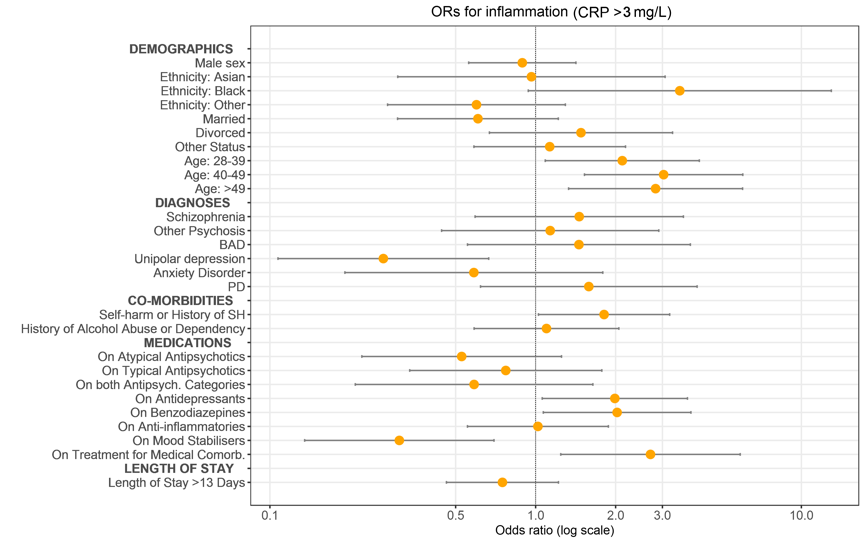


## Supplementary references

Cambridgeshire County Council Research and Performance Team, 2013. Population, Housing and Employment Forecasts Cambridgeshire County Council, Cambridge, p. 48.

Cunningham, H., Tablan, V., Roberts, A., Bontcheva, K., 2013. Getting more out of biomedical documents with GATE's full lifecycle open source text analytics. PLoS Comput Biol 9(2), e1002854.

Pearson, T.A., Mensah, G.A., Alexander, R.W., Anderson, J.L., Cannon, R.O., Criqui, M., Fadl, Y.Y., Fortmann, S.P., Hong, Y., Myers, G.L., 2003. Markers of inflammation and cardiovascular disease. Circulation 107(3), 499-511.

Ridker, P.M., 2003. Clinical application of C-reactive protein for cardiovascular disease detection and prevention. Circulation 107(3), 363-369.
